# Erratum zu: Demenzsensibilität in Akutkrankenhäusern: Warum die Umsetzung so schwierig ist, und wie sie dennoch gelingen kann

**DOI:** 10.1007/s00391-019-01667-4

**Published:** 2020-02-19

**Authors:** Sabine Kirchen-Peters, Elisabeth Krupp

**Affiliations:** Institut für Sozialforschung und Sozialwirtschaft e. V., Trillerweg 68, 66117 Saarbrücken, Deutschland

**Erratum zu:**

**Z** **Gerontol Geriat 2019**

10.1007/s00391-019-01631-2

Der Artikel „Demenzsensibilität in Akutkrankenhäusern“ von Sabine Kirchen-Peters und Elisabeth Krupp wurde ursprünglich am 18.10.2019 ohne „open access“ online auf der Internetplattform des Verlags publiziert. Die Autorinnen haben sich jedoch nach der Veröffentlichung in Bd. 52, Supplement 4, S. S291–S296 für eine „Open-access“-Veröffentlichung entschieden. Das Urheberrecht des Artikels wurde deshalb im Februar 2020 in © Der/die Autor(en) 2020 geändert. Der Artikel wird nun unter den Bedingungen der 4.0 International (https://creativecommons.org/licenses/by/4.0/deed.de) veröffentlicht, welche die Nutzung, Vervielfältigung, Bearbeitung, Verbreitung und Wiedergabe in jeglichem Medium und Format erlaubt, sofern Sie den/die ursprünglichen Autor(en) und die Quelle ordnungsgemäß nennen, einen Link zur Creative-Commons-Lizenz beifügen und angeben, ob Änderungen vorgenommen wurden.

